# An *ABCD1* Mutation (c.253dupC) Caused Diverse Phenotypes of Adrenoleukodystrophy in an Iranian Consanguineous Pedigree

**DOI:** 10.4172/1747-0862.1000222

**Published:** 2016-06-19

**Authors:** Masoud Mehrpour, Faeze Gohari, Majid Zaki Dizaji, Ali Ahani, May Christine V. Malicdan, Babak Behnam

**Affiliations:** 1 Department of Neurology, Firoozgar Hospital, Iran University of medical sciences (IUMS), Tehran, Iran; 2 Medical Student Research Committee (MSRC), Faculty of Medicine, Iran University of medical sciences (IUMS), Tehran, Iran; 3 Department of Medical Genetics, School of Medicine, Tehran University of Medical Sciences (TUMS), Tehran, Iran; 4 Department of Medical Genetics and Molecular Biology, Faculty of Medicine, Iran University of Medical Sciences (IUMS), Tehran, Iran; 5 NIH Undiagnosed Diseases Program, National Human Genome Research Institute (NHGRI), National Institutes of Health, Bethesda, Maryland, USA

**Keywords:** *ABCD1*, Adrenoleukodystrophy, X-linked, Mutation

## Abstract

**Objectives:**

Current study was the first to report a consanguineous Iranian pedigree with *ABCD1* mutation.

**Methods:**

Targeted molecular analysis was initially performed in three affected individuals in one family suspected to have X-ALD due to chronic progressive spasticity. Upon confirmation of genetic diagnosis, further neurologic and genetic evaluation of all family members was done.

**Results:**

A mutation in *ABCD1* was identified in 35 affected individuals (out 96 pedigree members). The c. 253dup, in exon 1, leads to a frame shift and a premature stop codon at amino acid position 194 (p.Arg85Profs*110). Surprisingly, affected individuals in our cohort show some variability in phenotype, including childhood cerebral ALD, adrenomyeloneuropathy, and addison-only disease phenotypes, expanding the phenotype of X-ALD with p.Arg85Profs*110.

**Conclusion:**

This report characterizes the clinical spectrum of an expanded Iranian pedigree with X-ALD due to an *ABCD1* mutation. Given a high frequency of carriers in this region, we expect the prevalence of X-ALD to be higher, underscoring the importance of genetic counseling through reliable identification of heterozygous as well as homozygote females in consanguineous communities.

## Introduction

X-linked adrenoleukodystrophy (X-ALD) (MIM 300100) is the most common inherited peroxisomal neurodegenerative disease. It presents with great phenotype variability [1], including seven and five male and five female different phenotypes, respectively [1,2]. In more than 75% of all ALD cases, childhood cerebral ALD (ccALD) and adrenomyeloneuropathy (AMN) are the most common subtypes. ccALD, which occurs in childhood, typically has a fatal course within 3-5 years after the onset of neurologic symptoms, while AMN is observed between the ages of 20 to 50 years and usually leads to progressive spastic paraparesis [3-6]. The impact on the quality of life is tremendous due to the high mortality rate of ccALD in the first decade of life, and the inability of patients with AMN to be economically productive. Adolescent and adult cerebral ALD (CALD), Olivo-ponto-cerebellar (OPC), asymptomatic, and Addison-only disease (AD) are among less frequently described forms [1].

All patients with X-ALD have mutations in *ABCD1* [7], thus *ABCD1* is still one of the most important candidate genes for ALD. To date, nearly 700 *ABCD1* mutations have been reported (“X-ALD database [http://www.x-ald.nl],” 2013). *ABCD1* consists of 10 exons that spans 19 kilobases (kb) of genomic DNA and produces a mRNA of 3.6 kb (NM_000033); it encodes ATP-binding cassette subfamily D, member 1 (*ABCD1* or ALDP), which is composed of 745 amino acids (NP_000024). *ABCD1* is a peroxisomal membrane ABC transporter that mediates transport of very long-chain fatty acids (VLCFAs; ≥ C22) across the peroxisomal membrane.

Defects in *ABCD1* leads to impaired peroxisomal beta-oxidation of VLCFAs, which is reduced to about 30% of control levels [8-10] in X-ALD patients. A subsequent accumulation of pathognomonic amounts of saturated VLCFAs occurs in plasma and some other tissues, including the brain white matter, the spinal cord, and adrenal cortex, as well as skin fibroblasts [11,12]. Increased plasma VLCFA level provides a reliable diagnostic tool for affected male identification. In 0.1% of affected males, however, plasma VLCFA levels are borderline and in addition, female obligate carriers can have false-negative results in about 20% [13]. Therefore, mutation analysis seems to be the best reliable approach for a genetic diagnosis.

In the present study, we report an *ABCD1* mutation with diverse X-ALD clinical manifestations in a big consanguineous Iranian pedigree, and highlight the importance of genetic screening before any pregnancy in asymptomatic women whose carrier status is unknown.

## Methods and Materials

### Patient selection and study protocol

In the present study, we reported an expanded Iranian pedigree with high consanguineous marriage rate and X-ALD involvement in Borujerd city (the capital of Lorestan province), Iran. In three affected members of the core family, direct sequencing revealed a variant in the first exon of *ABCD1* which raised the suspicion of ALD in other relatives. To screen probands' relatives and ancestors for their neurologic manifestations and from genetic point of view, we travelled to Borujerd city in August 2012. Due to geographical distribution of ALD, ethnicity would play an important role where all pedigree members belonged to Lorestan ethnicity in the study. Blood samples were taken from all family members, which were then subjects for leukocyte isolation. Genetic analysis was conducted as followed. Ancestry was determined and confirmed by relatives self-report. ALD definite diagnosis was based on the genetic analysis and sequencing results.

The study has been approved by the Iran University of Medical Sciences' institutional review board. The protocol was in accordance with the ethical principles of the Helsinki Declaration and an oral informed consent has been received from all study individual.

### *ABCD1* gene analysis

Genomic DNA was extracted from peripheral leukocytes using standard method [14]. The coding exons and the intron-exon boundaries of the *ABCD1* gene were amplified via PCR; primers and conditions were presented in Table 1.

Single-strand sequencing was performed utilizing gene specific primers and standard methods on an ABI 3730 (Applied Biosystems, Macrogen, South Korea). Sequences of all amplicons were compared with the published template (accession no. NM_000033) using Mutation Surveyor (version 3.20; SoftGenetics, State College, PA). Any changes in the sequence were checked against published polymorphisms and mutations and for conservation across species.

## Results

### Clinical manifestations

In this study, we studied 96 members of a pedigree whose affected members presenting with various clinical manifestations of X-ALD. The patients, including 51 females and 45 males with 3 to 90 years of age, were examined either in Borujerd (Lorestan province, Iran), or at the neurology department of the Firoozgar University Hospital. Clinical and molecular data are provided in Table 2. Three of the affected individuals were admitted in Firoozgar University Hospital, Tehran.

**Figure 1:**
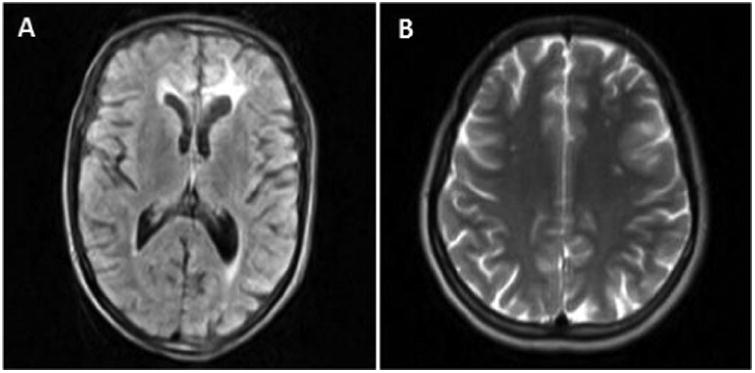
Brain MRI. Brain MRI of V-29 shows white matter hypersignal changes in FLAIR (A) and T2-weighted images (B), especially in bilateral fronto-parietal, periventricular, and centrum semiovale areas.

The proband (V-29) is a twenty one-year-old woman with a history of spastic gait for 8 months. She complained of urge urinary incontinence and frequency. An initial clinical diagnosis of multiple sclerosis (MS) was given. Neurological examinations showed hyper-reflexia, spasticity, and mild lower limbs weakness. Her brother was diagnosed with X-ALD, while her father (IV-19) was diagnosed with MS. Electromyography revealed apparently normal or decreased firing rate consistent with upper motor neuron lesion. Brain MRI T2 and FLAIR images showed diffuse multiple hyperintense lesions bilateral fronto-parietal, periventricular, and centrum semiovale (Figure 1). She was discharged with Baclofen and Tizanidine.

The proband's father, (IV-19) is 47 years old and had a long clinical diagnosis of and treatment for MS. For the past 8 years he had gait disturbance, imbalance, unilateral left lower limb weakness that progressively involved right lower limb, and urinary retention for 18 months due to sphincter dysfunction that necessitated clean intermittent catheterization for a year prior to his admission. Neurological examination revealed upper motor neuron signs (positive Babinski, increased deep tendon reflex, clonus, and spastic tone), neurogenic bladder, emotional incontinence, and pseudobulbar affect. He was also noted to have hyperpigmentation in mouth and skin. He had a son with ALD, a daughter (Case V 29) with spastic paraparesia initially diagnosed to have MS, but eventually diagnosed with X-ALD. He also had a daughter (V-28) with ophthalmic and gait disturbances who succumbed to death at which was expired in her 11-years of old age. He also reported some similar signs in his siblings.

The proband's uncle (IV-9), a 41-year-old man with spastic paraparesia, was seen Firoozgar University Hospital due to progressive lower limb weakness which started at the age of 10 years, and gait disturbance at 39 years of age. He also suffered from urge incontinence and urinary frequency. Plantar reflexes were up bilaterally.

We proposed X-linked adrenoleukodystrophy as the most possible diagnosis for these patients with respect to clinical manifestations, image findings, and their family history. These led us to perform a genetic study to confirm the diagnosis. We found that among 96 members of the pedigree, nineteen (fifteen male and four female) were affected and sixteen female were carriers. Consequently, high consanguinity rate in mentioned Iranian pedigree showed involvement of 19 in 96 pedigree members (about 20%), while the proportion is 1 in every 17,000 individual worldwide [15]. Comparing the two prevalence rates indicates that X-ALD was significantly more frequent in our studied Iranian pedigree (Odds Ratio 4249.7500, 95% CI 224.6575 to 80390.7075, p-value< 0.0001) than what was previously reported.

### Molecular analysis of *ABCD1*

**Figure 2:**
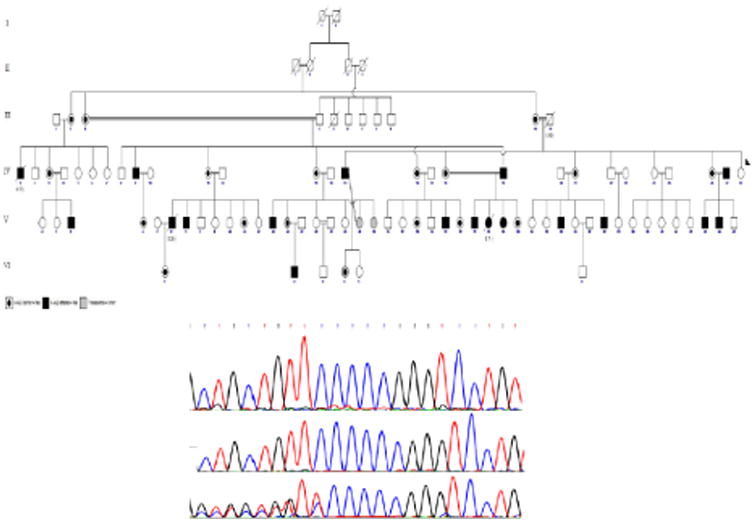
Pedigree and molecular diagnosis. (A) The cross inheritance of recessive X-linked adrenoleucodystrophy alleles in the Iranian consanguineous pedigree. (B) First above row indicates normal genotype. Second row represents a homozygous mutant sequence, while the third row represents heterozygote genotype.

Sequencing analysis showed that the cases including patients were either homozygous females, or hemizygote (X-linked) males, or heterozygote female carriers for c.253dup in exon 1 and predicted to result to a premature stop codon at amino acid position 194 (p.Arg85Profs*110). Through genetic analysis of the gene, the mutation has been confirmed in 35 cases consist of thirteen (7 affected males and 3 affected females, and three male carriers) with some neurologic symptoms and twenty (7 affected male and 2 affected females, and 11 female carriers) patients without any neurologic manifestations, respectively. Three patients did not let physician examine for their symptoms (two carrier female encoded as III2 and IV3, and an affected male, V3). The pedigree and chromatograph, showing involved probands' hemi/homozygocity and also the detected mutation, are presented in Figure 2.

We also submitted the newly recognized HGVS, entitled as NM_000033.3:c.253dup, in ClinVar database, NCBI, with SCV accession number of SCV000196515, RSV accession number of RCV000149556, and Organization ID of 505319 (Gohari, 2014).

## Discussion

X-ALD is the only disease associated with *ABCD1* gene [7,16]. Thus far, 751 *ABCD1* gene mutations have been listed in X-ALD database (“X-ALD database [http://www.x-ald.nl],” 2013). Mutations are scattered throughout the entire coding region; reports are usually infrequent and confined to a single study. Some regions of *ABCD1* are considered to be hot-spots in particular ethnic groups; while most mutations are in exon 1, exons 5 and 6 are most commonly involved in Caucasians and Chinese, respectively [17-19].

All the affected individuals in our cohort have the same recurrent inherited mutation. Although this mutation has been reported five times independently, none of these reports included large sample size, or provided detailed clinical characteristics and rare manifestations. Nevertheless, it would seem that this region may be a potential hot-spot [1,20-23].

Although there had been no systematic study conducted to support the predicted structure and function of ALDP, previous studies showed that missense mutations in *ABCD1* leading to decrease in ALDP levels may interfere with the peroxisomal targeting mechanisms of the newly synthesized ALDP molecules, their correct membrane insertion, and their correct folding. The c.253dup results in a premature truncation of the protein (p.Arg85Profs*110) and therefore can be predicted to affect *ABCD1* abundance; it is thus plausible that our mutation, similar to other truncating mutations in *ABCD1*, can result to the same defects of peroxisomal targeting as reported [24] and suggest that the c-terminus might be necessary for its proper function.

Interestingly, we observed two major forms of the X-ALD: childhood cerebral ALD and adrenomyeloneuropathy. We also have one affected male with lower limb hyperreflexia without any other neurologic manifestations who showed some degrees of adrenal insufficiency (Addison-only disease). The range of phenotypic expression, its severity, and the prognosis of males versus females' involvement were unpredictably variable, even with the same mutation, suggesting the contribution of confounding factors yet to be identified, although X-ALD has been regarded as a monogenic disorder. Nonetheless, environmental factors, head trauma, inflammation, and altered immune responses have been implicated as risk factors for cerebral ALD form. Other factors implicated include latitude, environmental modifiers varying with latitude (e.g. ultraviolet radiation), and factors affecting variable gene penetrance/expressivity [25]. Modifier genes, epigenetic, or stochastic factors can also be involved in highly variable clinical manifestations and lack of any established genotype-phenotype correlation [13,26-30]. A continued longitudinal and genetic study in large pedigrees will help identify these confounding variable for phenotype variability.

Interestingly we also observed that four females (two carriers, and two genetically unaffected) (Table 2) suffered from β-thalassemia minor. This observed concordance is not new, and some rare cases with both X-ALD and β-thalassemia has already been [31], giving emphasis to the need for a mandatory careful screening program of known single gene disorders among consanguineous couples. Some modern and advanced technologies in the genetics field, including different formats of next generation Sequencing, will be valuable and cost-effective in getting information for genetic counseling [32-35].

One of the affected individuals in our cohort (V-28) had ophthalmic manifestations; she died at 11 years of age before our screening, so was missed for further evaluation. Eye involvement is among rare manifestations of ALD; this may be the case as *ABCD1* expression in the eye is very low; in Expressed Sequence Tag (EST) profile, the proportion of “gene EST/total EST in pool” equals to 3/208840 and contains 14 transcript per million (“EST profile,”)(“EST profile,”)(“EST profile,”)(“EST profile,”)(39).

Lifespan of the affected individuals in our cohort also are variable. There were three affected males who died at the age of 11 (V-43), 15 (IV-1), and 26 (V-6) years. The latter (V-6) had AMN phenotype; death could have presumably due to progressive cerebral demyelination which is observed only in 20% of AMN patients (“X-ALD database [http://www.x-ald.nl],” 2013). ; V-43 did not respond to stem cell transplantation.

Our systematic clinical and genetic analysis has increased our molecular diagnostic rate, both in symptomatic and asymptomatic individuals. As patients can present with pure neurological symptoms, which mimic other more common neurological disorders, such as MS, neurologists must be alerted to include X-ALD in the differential diagnosis of demylelination. Studies have shown that X-ALD inflammatory demyelination can resemble those found in MS, the most common central nervous system demyelinative disease [29,30]. The high consanguinity rate in our cohort also highlights the importance of considering X-ALD diagnosis, especially in individuals belonging to Lorestan ethnicity, who presents with progressive upper motor neuron signs [17-19].

Early diagnosis is essential for personalized medicine, especially that bone marrow transplantation has been shown to be effective at the early stages of cerebral symptoms, which is usually noted in 50% of cases 10 years after the onset of disease [36-38]. The diagnosis usually is suspected by the detection of elevated VLCFA levels and increased ratios of C26:0/C22:0 and C24:0/C22:0 in fasting blood, although about 20% of the obligate heterozygotes usually give normal values. A combination of genetics diagnosis and VLCFA levels would be helpful in establishing early diagnosis.

The study had limitations, including lack of clinical information from three affected individuals (III-2, IV-3, and V-3). The complete clinical neurologic examination of the core family, however, was helpful in delineating differences in clinical presentation. Another limitation would be the lack of neuroradiological exam, which was only done in V-29. In the past, wide-ranged MRI pattern either on lesion location or isotopic diffusion/T2 pattern [5,39-41] were observed in X-ALD patients, and a strong association between the presence of contrast enhancement on T1-weighted MR images and X-linked ALD progression [42] has been identified.

In conclusion, this is the first major ALD report in Iranian population, Lorestan province. The study highlights how high consanguineous marriage rate can significantly raise the ALD prevalence and female involvement in comparison to communities with routine low rate or non-consanguineous marriage. Further studies on X-ALD prevalence, mutation patterns, modifier gene, epigenetic factors, environmental feature, and clinical course after bone marrow transplantation, will be needed to understand the pathophysiology of this devastating disease.

## Figures and Tables

**Table 1:** *ABCD1* primers.

Primer	Sequence (5′->3′)
*ABCD1* 1F	GAGCAACAATCCTTCCAGCC
*ABCD1* 1R	CCACACCTTTGGCATCAGCC
*ABCD1* 2F	ACTGGGAGACCCTGACCATC
*ABCD1* 2R	CTGAGTTGGGCCCTCGTGA
*ABCD1* 34F	CCATTTGCAGAAGAGCCTCG
*ABCD1* 34R	GCGGGAATAGGAGGAGCTGG
*ABCD1* 5F	GGCACGCAGACTCCCCAGAA
*ABCD1* 5R	TTGCCAGCACAGACAGGCG
*ABCD1* 67F	TCGGGCATTGGGAGCCTC
*ABCD1* 67R	TGGCACCTGGCACTTTAGAC
*ABCD1* 810F	GAGCCAAGACCATTGCCCTC
*ABCD1* 810R	AGGGGCGGGGTGCGTGCATG

**Table 2:** Clinical and genotypic data of the patients- 35 patients were screened for the mutation and 3 were died prior to the genetic confirmation. (AMN: Adrenomyeloneuropathy; ccALD: Childhood cerebral ALD).

No	Pedigree Code	Age	Sex	Genotype	Phenotype	Others
1	III-2	-	Female	Carriers	-	-
2	III-3	88	Female	Carriers	Asymptomatic	-
3	III-10	73	Female	Carriers	Asymptomatic	-
4	IV-3	-	Female	Carriers	-	-
5	IV-11	57	Female	Carriers	Asymptomatic	-
6	IV-13	60	Female	Carriers	Asymptomatic	-
7	IV-16	55	Female	Carriers	AMN	-
8	IV-18	43	Female	Carriers	AMN	-
9	IV-21	54	Female	Carriers	AMN	-
10	IV-26	36	Female	Carriers	Asymptomatic	-
11	V-4	7	Female	Carriers	Asymptomatic	-
12	V-11	28	Female	Carriers	Asymptomatic	-
13	V-14	29	Female	Carriers	Asymptomatic	Β-Thalassemia minor
14	V-30	5	Female	Carriers	Asymptomatic	-
15	VI-1	10	Female	Carriers	Asymptomatic	-
16	VI-4	16	Female	Carriers	Asymptomatic	Β-Thalassemia minor
17	V-23	32	Female	Affected	Asymptomatic	-
18	V-26	21	Female	Affected	Asymptomatic	-
19	V-28	Dead by 11 yr;	Female	Affected	ccALD	Dead before screening
20	V-29	22	Female	Affected	AMN	-
21	IV-1	Dead by 15 yr;	Male	Affected	ccALD	Dead before screening
22	IV-9	41	Male	Affected	AMN	-
23	IV-15	15	Male	Affected	AD	-
24	IV-19	57	Male	Affected	AMN	Primary diagnosis and treatment of MS
25	IV-27	36	Male	Affected	Asymptomatic	-
26	V-3	26	Male	Affected	-	-
27	V-6	Dead by 26 yr;	Male	Affected	AMN	Dead before screening
28	V-7	45	Male	Affected	Asymptomatic	-
29	V-13	23	Male	Affected	AMN	-
30	V-25	24	Male	Affected	Asymptomatic	-
31	V-27	21	Male	Affected	Asymptomatic	-
32	V-33	27	Male	Affected	Asymptomatic	-
33	V-36	24	Male	Affected	Asymptomatic	-
34	V-43	8	Male	Affected	ccALD	Treated with stem cells but dead by 11 yr
35	V-44	7	Male	Affected	Asymptomatic	-
36	IV-22	45	Male	Genetically unaffected	AMN	-
37	V-19	21	Female	Genetically unaffected	Asymptomatic	Β-Thalassemia minor
38	V-20	34	Female	Genetically unaffected	Asymptomatic	Β-Thalassemia minor

## Abbreviations

AMN: Adrenomyeloneuropathy
*ABCD1*: ATP-Binding Cassette Subfamily D, Member 1
ccALD: Childhood Cerebral ALD
CI: Confidence Interval
MS: Multiple Sclerosis
VLCFA: Very Long-Chain Fatty Acid
X-ALD: X-linked Adrenoleukodystrophy
